# Mechanical and Non-Destructive Testing of Plasterboards Subjected to a Hydration Process

**DOI:** 10.3390/ma13102405

**Published:** 2020-05-23

**Authors:** Zbigniew Ranachowski, Przemysław Ranachowski, Tomasz Dębowski, Adam Brodecki, Mateusz Kopec, Maciej Roskosz, Krzysztof Fryczowski, Mateusz Szymków, Ewa Krawczyk, Krzysztof Schabowicz

**Affiliations:** 1 Experimental Mechanics Division, Institute of Fundamental Technological Research, Polish Academy of Sciences, Pawińskiego 5B, 02-106 Warszawa, Poland; zranach@ippt.pan.pl (Z.R.); pranach@ippt.pan.pl (P.R.); tdebow@ippt.pan.pl (T.D.); abrodec@ippt.pan.pl (A.B.); mkopec@ippt.pan.pl (M.K.); 2 Faculty of Mechanical Engineering and Robotics, AGH University of Science and Technology, Aleja Mickiewicza 30, 30-059 Kraków, Poland; mroskosz@agh.edu.pl; 3 Faculty of Energy and Environmental Engineering, Silesian University of Technology, ul. Akademicka 2A, 44-100 Gliwice, Poland; krzysztof.fryczowski@polsl.pl; 4 Faculty of Civil Engineering, Wrocław University of Science and Technology, Wybrzeże Wyspiańskiego 27, 50-370 Wrocław, Poland; Mateusz.Szymkow@pwr.edu.pl (M.S.); krawczyk.em@gmail.com (E.K.)

**Keywords:** plasterboards, moisture content, hydration processes, mechanical properties, ultrasound measurements

## Abstract

The aim of this study was to investigate the effect of plasterboards’ humidity absorption on their performance. Specimens’ hydration procedure consisted of consecutive immersing in water and subsequent drying at room temperature. Such a procedure was performed to increase the content of moisture within the material volume. The microstructural observations of five different plasterboard types were performed through optical and scanning electron microscopy. The deterioration of their properties was evaluated by using a three-point bending test and a subsequent ultrasonic (ultrasound testing (UT)) longitudinal wave velocity measurement. Depending on the material porosity, a loss of UT wave velocity from 6% to 35% and a considerable decrease in material strength from 70% to 80% were observed. Four types of approximated formulae were proposed to describe the dependence of UT wave velocity on board moisture content. It was found that the proposed UT method could be successfully used for the on-site monitoring of plasterboards’ hydration processes.

## 1. Introduction

The hydration of calcium sulphate hemihydrates (stucco reaction) nowadays has great industrial and economic importance. Based on the data published by Eurogypsum [1], ca. 5 million tonnes of these products are used every year in the European building industry. The plasterboards are used in renovations, as well as for building constructions. They could be used for both, humidity- and fire-resistant applications. Humidity-resistant boards are usually reinforced with glass fiber mesh. In the novel method presented in [2], nano silver protecting coating was described to enhance the plaster performance. On the other hand, fireproofing panels used for building fire protection systems, usually have low water content and silica fillers in their structure. The article [3] presents an example of silica filler application in shrinkage controlling and, consequently, reducing cracks. Novel, synthetic gypsum boards were characterized by their enhanced performance. This by-product was described and tested in [4]. Despite years of research, a fully convincing description of the stucco hydration reaction was not presented. The conversion of hemihydrate (CaSO_4_·0.5H_2_O) to gypsum (CaSO_4_·2H_2_O) was studied mainly through quenching reactions or indirect methods [5,6]. Additionally, the CaSO_4_-H_2_O system could have up to five phases [7]. Hemihydrates, prepared from the dehydration of gypsum, exist in two forms—*α* and *β*. The most popular *β*-form is generated via dry calcining (120–180 °C). The *α*-form is prepared under hydrothermal conditions, involving high pressure—up to 8 bar. Both forms were described as structurally undistinguishable, but with different crystal habits [8]. *α*-stucco is much more expensive and difficult to generate. It is used wherever mechanical strength is of prime importance. The much less expensive *β*-form is produced and applied on a considerably larger scale. *β*-stuccois widely used in the production of plasterboards. Gypsum product is similar for both stucco forms from which is it produced; however, the reaction process is different. *α*-stucco induction time is shorter and conversion percentage is higher. It should be emphasized that the reaction never occurs with 100% efficiency. In order to increase the reaction rate and improve the hardness of the product, gypsum seed crystals are usually added to the stucco and water mixture. This does not affect the amount of water needed. In the case of low concentrations of seeds, the rate of crystal growth is greater than the rate of nucleation. When concentrations of added seeds are higher (above 0.5% *w/w*), the situation is reversed and nucleation is faster [9]. The stages of the hydration process are well recognised—a comparatively rapid dissolution of hemihydrate and a subsequent, slower precipitation of CaSO_4_·2H_2_O [10]. Nevertheless, description of the kinetics and mechanism of the whole hydration process was not fully investigated yet. It is known that the plasterboards are made of partially dehydrated gypsum of high porosity (5% ÷ 40% by vol.) so its density is low but the mechanical strength remains at the acceptable level. The noteworthy physical effect caused by the further unwanted hydration of the plasterboard volume is the loss of their elasticity and strength. Plasterboards products are exploited in the environment with increased humidity, which reduces their durability significantly. Additionally, the occurrence of organic mould growth in hydrated plasterboards might create severe health issues.

The following papers were devoted to testing procedures of plasterboards. During the inspection and diagnostics of surfaces made of gypsum boards, the method presented in reference [11] was found to be useful. This study presents a data system supporting the inspection and diagnostics of partition walls and wall cladding assembled using the drywall construction method. Furthermore, the classification of anomalies and their probable causes was described. In reference [12], the system supporting the inspection and diagnostics of partition walls was described and additional research on the correlation matrix was supplemented. In [13], the physical and mechanical properties of innovative panels were determined. These energy-saving panels were successfully used in India. In addition to traditional panels, composites are increasingly appearing in building constructions. The experimental testing of the compressive strength of gypsum wall panels was described in [14] and the physical and mechanical properties of panels were determined. The above article, as well as [15], describes composite panels consisting of gypsum, plaster and fiberglass. Currently, the world’s awareness of society is growing more and more frequently, apart from the properties described in the above articles, the materials must be ecological. In [16], a recycling process for gypsum plasterboard was presented. It comprised of grinding and calcining the residue of drywall sheets and further resulted in the obtaining of the product of acceptable quality. It was concluded that, after rehydration, it was possible to use only the gypsum waste to mould solid specimens. This experiment showed that product merely discarded in the environment could be also recycled. The influence of humidity on the properties of concrete was investigated by using contact and non-contact methods and was reported in [17], but, so far, no similar research in relation to plasterboards was performed.

## 2. Materials and Methods 

Five sets of specimens, six pieces of each, made of five different plasterboards types (labelled A, B, G, I and K) were prepared for the examination. All the specimens were 12.5 mm thick. The following board types were investigated:

- A—Standard board for indoor installations;
- B—Low-quality, standard-type board rejected after quality control by the producer;
- G—Fire-resistant type;
- I—Acoustic-insulating type;
- K—Humidity-resistant type.

**Figure 1:**
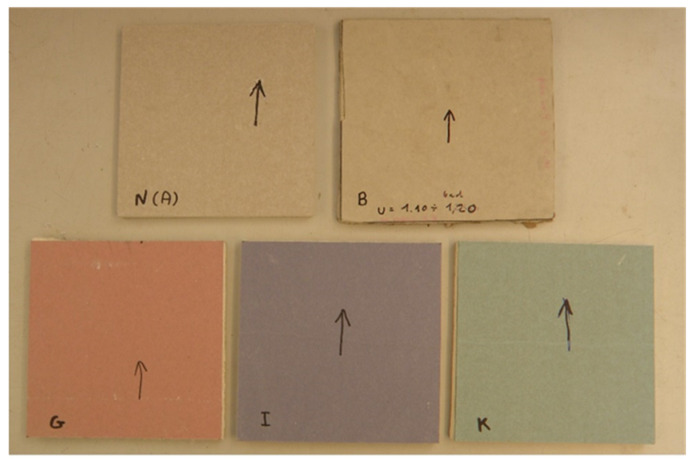
View of five types of square 200 × 200 mm plasterboard specimens prepared for examination.

Figure 1 shows the general view of five types of square 200 × 200 mm plasterboard specimens, prepared for examination. The black arrows on the board surfaces denote the fabrication direction.

The microstructural observations of all types of investigated boards were analysed by using optical light microscopy (AM4113ZTL 1.3 Megapixel Dino-Lite Digital Microscope with integral LED lighting, (Keyence, Mechelen, Belgium) and a low vacuum scanning electron microscope JEOL JSM6460 LV (Jeol, Tokyo, Japan) with an Energy Dispersive Spectroscopy (EDS) analyser, fabricated by JEOL. 

The following experimental procedure was performed to assess the deterioration of mechanical properties due to the hydration process. The specimens made of five types of plasterboard were initially subjected to mechanical testing in the as-received state. The other set of specimens was immersed in water at room temperature for 10 min, dried at the same temperature for 60 min and finally directed to mechanical testing. The third set of specimens was subjected to the hydrating procedure twice and then the flexural test was performed. Applying the procedure described above, the three levels of hydration were examined: (1) dried in standard room conditions, (2) hydrated with lower intensity and (3) hydrated with higher intensity. The attempts to repeatably introduce the different states of hydration were not successful. The controlling of the specimens’ water intake was performed by consecutive weighting.

The method of moisture content measurement was performed as follows. The plasterboard specimens in the as-received state were dried for 1 hour, applying a hot air fan, and stored at room temperature in dry conditions for 24 hours. Then, the specimens were weighted and their mass in dry state *m*_0_ was determined. The mass in hydrated state *m_h_*_1_ and *m_h_*_2_ was determined 60 min after 10 min of hydration (or, after two cycles of hydration). The moisture content m_c1_ after one cycle of hydration and the moisture content *m_c_*_2_ after two cycles of hydration were given by the following formulas:

*m_c_*_1_ = (*m_h_*_1_ − *m*_0_) · 100/m_0_;  *m_c_*_2_ = (*m_h_*_2_ − *m*_0_) · 100/*m*_0_ (1)

**Figure 2:**
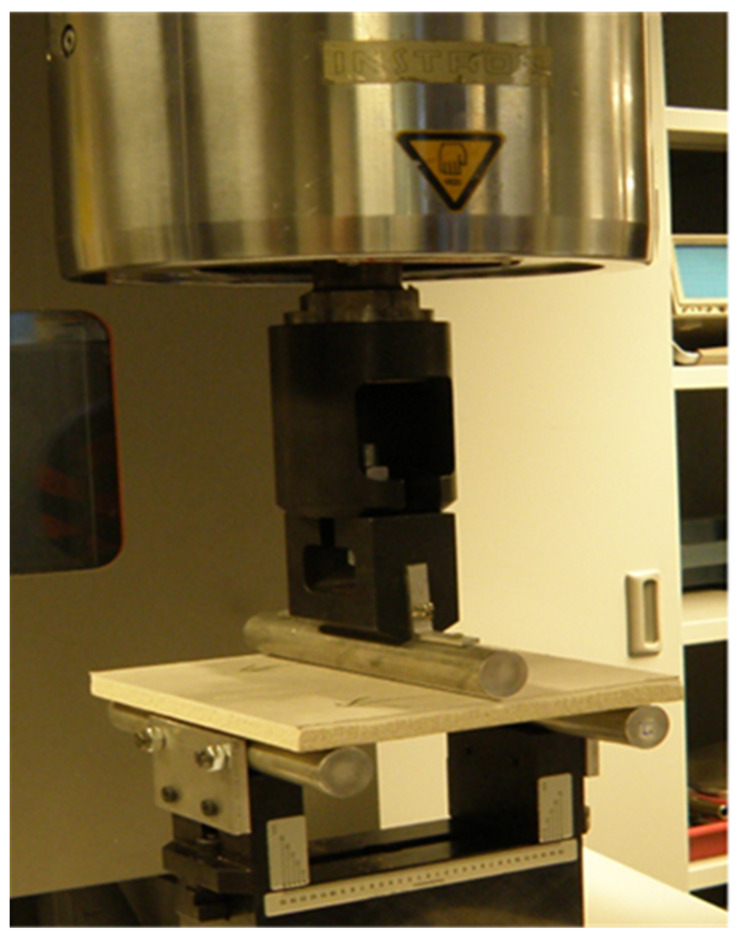
Experimental setup for the 3-point bending test of plasterboards.

The A-type boards have been designed for usage in the compartments where the relative humidity did not exceed 70%. The usage of the K-type boards in the increased humidity environment (up to 85%) was allowed within a period not exceeding ten hours. Gypsum plasterboards were composed of a plaster core encased in and firmly bonded to outer paper liners. Due to its fabrication method, the lining and also the board as a whole showed significant anisotropic properties [17,18,19,20]. Therefore, the mechanical properties of plasterboard sheets were determined in parallel and perpendicular to the sheet production direction, applying standard EN 520 [21]. Conventionally, a three-point bending test of plasterboards is the established procedure for their mechanical properties assessment. Square-shaped specimens were tested in this research. The procedure consisted of the loading of two specimens sets, two pieces each, in two directions mentioned above. The flexural strength of plasterboard was defined at maximal force registered by the loading system during the three-point bending. The bending test of investigated specimens was performed by using a hydraulic MTS-858 testing machine (MTS, Eden Prairie, Minnesota, USA) of 250 kN capacity, as was shown in Figure 2. The maximal force registered by the loading system was recalculated to the stress applied to the specimens, and, therefore, at 100 N of load, corresponded with 750 kPa of stress. The investigated material was brittle and had a porosity of 20%–40%. Therefore, the measurements of the modulus of elasticity were highly dispersed and not reliable.

Ultrasonic longitudinal wave velocity c*_L_* was determined in all specimens to find the dependence of wave velocity on the degree of hydration. The specifications of the investigated specimens are shown in Table 1 and Table 2.

In this study, a new, non-destructive method of the plasterboard hydration assessment was proposed. The selection of such a method was based on research presented in [22,23], where the deterioration of material elasticity in different building material, including the brittle matrix with air pores, were determined by using the ultrasound testing (UT) method. The dilatation of UT wavelength measured in the investigated material was, in any case, less than 2 mm, which is at least six times smaller than the material thickness. This was the main reason for the propagation of the longitudinal wave model adoption and neglection of the possible Lamb wave. Therefore, the change of UT longitudinal wave velocity *c_L_* was proposed to be a measure of plasterboard mechanical properties deterioration due to occurring hydration. In large-scale objects of small thicknesses, such as the plasterboards, the following dependence of UT longitudinal wave velocity *c_L_* and the scalar modulus of elasticity *E* could be used:

(2) $$c_{L}=\sqrt{\frac{E}{\rho \left(1-\nu^{2}\right)}}\;$$

where *ρ*—bulk material density; ν —Poisson ratio.

The dependence of c*_L_* on concrete humidity was investigated by using contact and non-contact methods [24]. The UT waves’ velocity determined by the authors remained in the range of 3500–4500 m/s. It was found that, in concrete, *c_L_* increases alongside increases in humidity storage. No remarkable loss of the concrete elasticity modulus due to humid conditions of its storage was observed. However, in highly porous and moisture-sensitive gypsum, a decrease in elasticity modulus and mechanical strength occurred with increased humidity content. Subsequently, a decreasing *c_L_* was found. This tendency was further examined in presented paper. Due to the high attenuation of UT waves in gypsum (2 to 5 dB per mm of thickness) and its lower specific density in relation to concrete, the authors have applied a dedicated instrumentation set to c*_L_* measure in this material. c*_L_* was determined with the accuracy higher than 0.5%, measuring the time of propagation *T* of the elastic wave front across the board of known thickness *d* with application of the formula *c_L_* = *d/T*. The investigation was performed by using the computerized ultrasonic material tester UTC110, produced by Eurosonic (Vitrolles, France) [25]. It was reported in [24] that low-frequency ultrasound (200–600 kHz) is reasonable for the testing of building materials, where the specimens’ thicknesses do not exceed several centimeters. However, such low frequency range ultrasound cannot be used for the testing of millimeter-thick boards due to excessively long wavelength and further errors in *c_L_* measurement. The series of preliminary tests performed by the authors have revealed that the required sensitivity of ultrasound parameters to investigate the structural properties of gypsum boards was achieved when the ultrasonic wavelength is comparable to the dimensions of local voids and pores. This wavelength *λ* reminded in the following relation to the frequency *f* of emitting source and propagation velocity c_L_ of travelling ultrasonic waveform:

(3) $$\lambda =\frac{c_{L}}{f}$$

**Figure 3:**
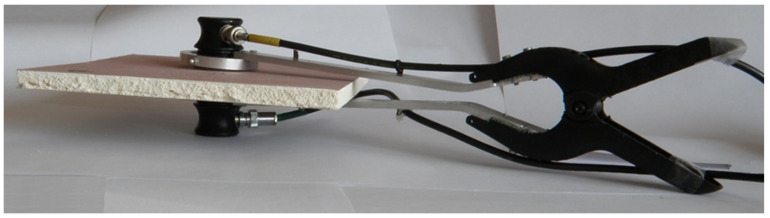
Detailed view of the custom designed holder for correct coupling between the ultrasonic transducers and both sides of the plasterboard specimen.

Thus, taking into consideration the propagation velocity of 900 ÷ 1800 m/s registered in plasterboards, the authors recommend the application of frequency of 1 MHz to achieve the propagation of waveforms in the range of 0.9–1.8 mm. The instrumentation used for measuring included transmitting and receiving transducers of type Videoscan, fabricated by Olympus [26]. These transducers allow one to emit the ultrasonic beam of 19 mm in diameter at 1 MHz frequency. That modern transducer type was designed for coupling with low-density (i.e., 600–2000 kg/m^3^) materials and thus exhibiting a low acoustic impedance of 10 MegaRayl. The contact between the rough surface of the board and the transducers’ face was achieved by using 0.6-mm-thick polymer jelly interfacing foil (Olympus PM-4-12, US Division of Olympus, Waltham, Massachusetts, US. The investigation was not aimed to recover particular defects in the specimens. Therefore, a relatively wide penetrating UT beam was used. The use of higher intensity of the beam was achieved to suppress the remarkable attenuation of the material. The custom-designed holder with articulated joints and compressing spring was prepared for the correct coupling of the transducers and investigated board surface. The detailed view of the holder is presented in Figure 3.

## 3. Results and Discussion 

### 3.1. Microstructural Characterization of Plasterboards in the As-received Condition

**Figure 4:**
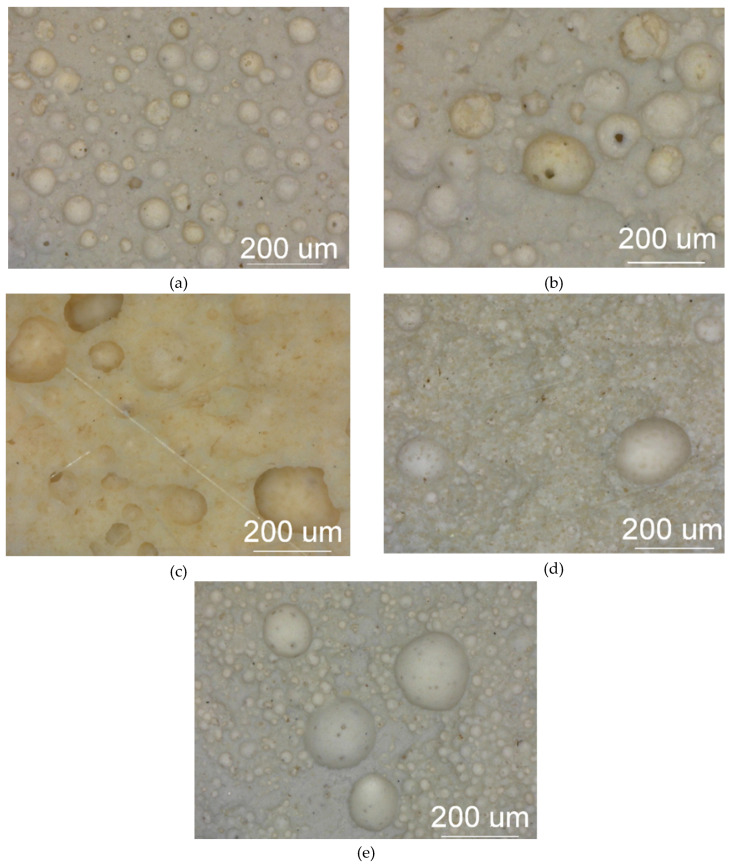
Macrostructure images of investigated specimen types: (**a**) type A, (**b**) type B, (**c**) type G, (**d**) type I, (**e**) type K.

The essential stage of plasterboard fabrication is a foaming process in which the porosity of gypsum increases up to a required level. This process is driven by the addition of the surface-active agents [9]. Such addition result in a formation of a complex structure with large, mostly spheroidal macropores, smaller pores of irregular shape, being interlaced with blade and needle shaped crystals of calcium sulphate. Most macropores were measured with ca. 100 µm of diameter; however, smaller and larger structures were also visible. Calcium sulphate crystals were only observed by using SEM. The distribution and size of the macropores were assessed by using optical microscopy. The microstructures of investigated specimen types are presented in Figure 4.

The dominant macropores with a diameter ranging from 20 to 200 µm were uniformly distributed in a significant volume of specimen derived from the standard plasterboard designed for indoor installations (Figure 4). This microstructure resulted in a low product density (648 kg/m^3^). 

The B-type specimen was characterized by small pores (10–20 µm) of irregular shape that appear in considerable quantity. These pores were also present in SEM micrographs. In both cases, they appeared as ‘black speckles’ because these regions do not reflect visual light nor electrons. Small pores situated at the walls of the foamed gypsum form the openings among the cavities. Such material behaviors modify the overall system of closed porosity into the open porosity system. This resulted in increased moisture permeability as well as in reduced UT longitudinal wave velocity. The microstructure of G-type, fire-resistant plasterboard was characterized by less regular shapes of macropores. The distribution of macropores was more sparse within the compact matrix, resulting in an increase in specific density (880 kg/m^3^). Specimens of type I and K were characterized by a considerably higher presence of smaller pores with diameters between 10 and 20 µm, immersed in the compact matrix with increased contents of very small gypsum crystals. This resulted in the remarkable growth of a specific density and a much higher compactness of these structures.

**Figure 5:**
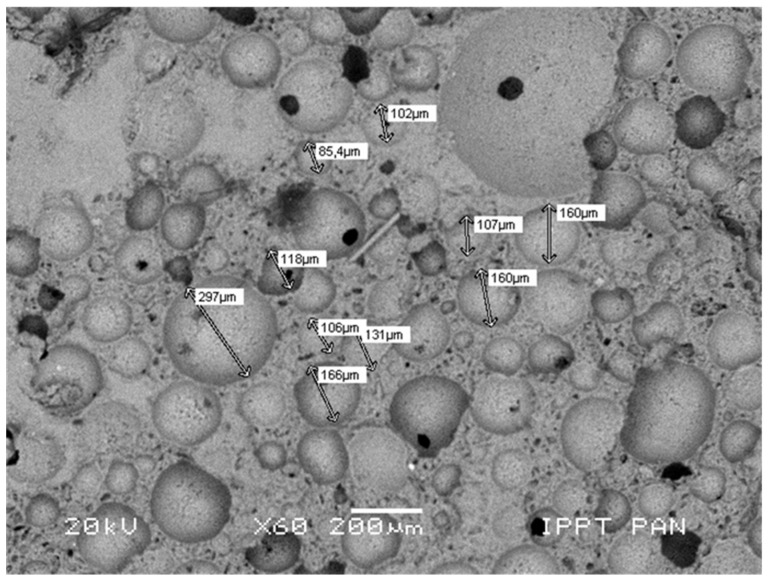
Microstructure of foamed gypsum matrix, with various sizes of pores.

Figure 5 shows a detailed view of a regular- and irregular-shaped pore system of a standard plasterboard core. The micrograph of the foamed gypsum matrix was made by using low vacuum SEM. The variety of pore sizes was presented.

**Figure 6:**
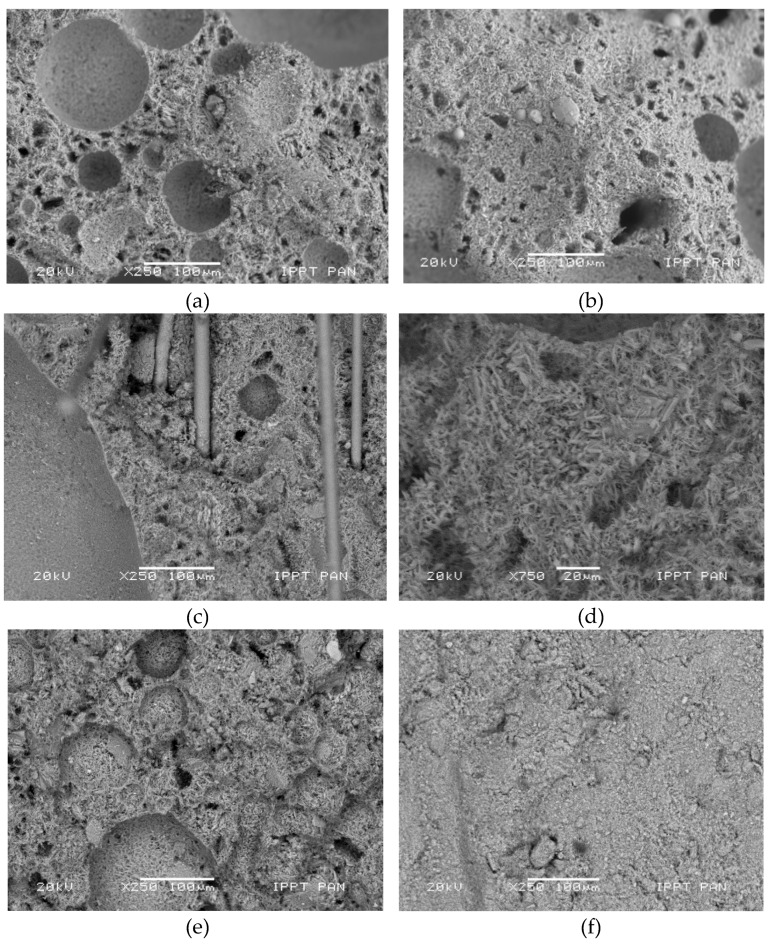
Microstructure of investigated specimens: (**a**) type A, (**b**) type B, (**c**) and (**d**) type G—low and high magnifications of the glass-fiber-reinforcement region—(**e**) type I, (**f**) type K.

Figure 6 presents the microstructure of investigated plasterboard types. Figure 6a presents a micrograph of a standard A-type plasterboard. The structure of this material consists of large macropores within the matrix of blade- and needle-shaped crystals. The volume of small, irregular-shaped pores is relatively low. The microstructure of the B-type specimen (Figure 6b) consists of the high number of irregular-shaped micropores within the open porosity system. The microstructure of the fire-resistant G-type plasterboard (Figure 6c) is generally similar to the standard one; however, it is more compact and it is reinforced with glass fibers. A reduced number of macropores occurred in acoustic insulating plasterboard I type (Figure 6d). Increased humidity penetration resistance was achieved through the large regions of high compact gypsum containing very small and densely spaced crystals, as presented in Figure 6f. The data presented in Table 3 include the weight percentage of elements for boards of type A and G. The mass percentage volume values, i.e., 55% wt. for oxygen, 19% wt. for sulphur and 23% wt. for calcium, presented a good agreement with theoretical stoichiometric values specified for CaSO_4_·2H_2_O. In the case of a gypsum molecule, these theoretical stoichiometric percent values for oxygen, sulphur and calcium were 55.8, 18.6 and 23.3, respectively. The enlisted data confirmed that the plasterboard cores were produced by using pure, technical gypsum raw material. Increased silicon content observed in the board of type G resulted from the presence of glass-fibre reinforcement. 

### 3.2. The Effect of Hydration on the Mechanical Properties of Plasterboards

**Figure 7:**
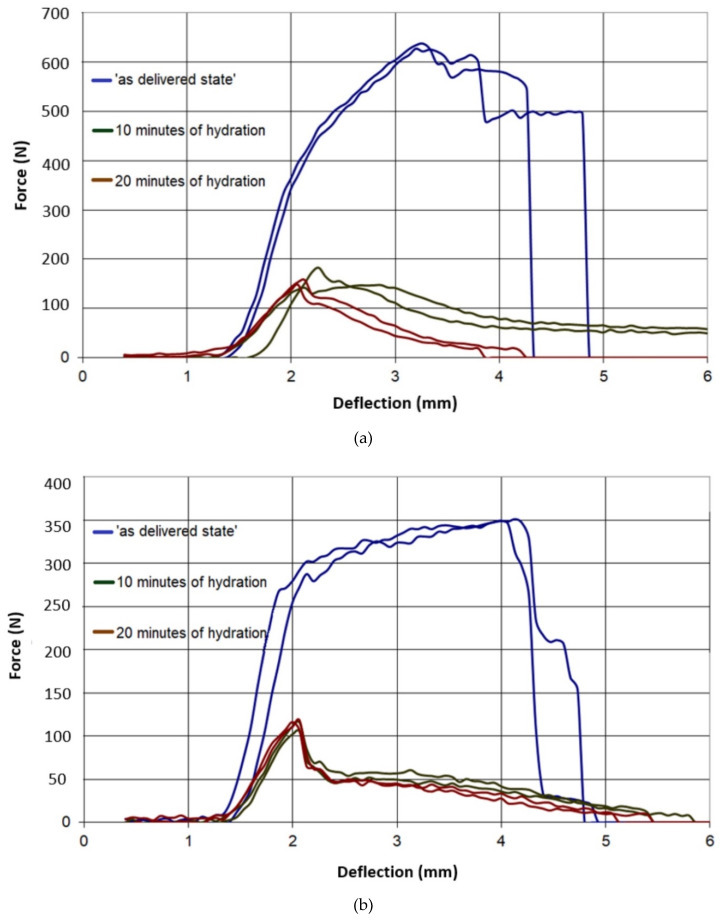
Force–deflection curves of the tested specimens of type A, (**a**) parallel to the sheet production direction, (**b**) perpendicular to sheet production direction.

**Figure 8:**
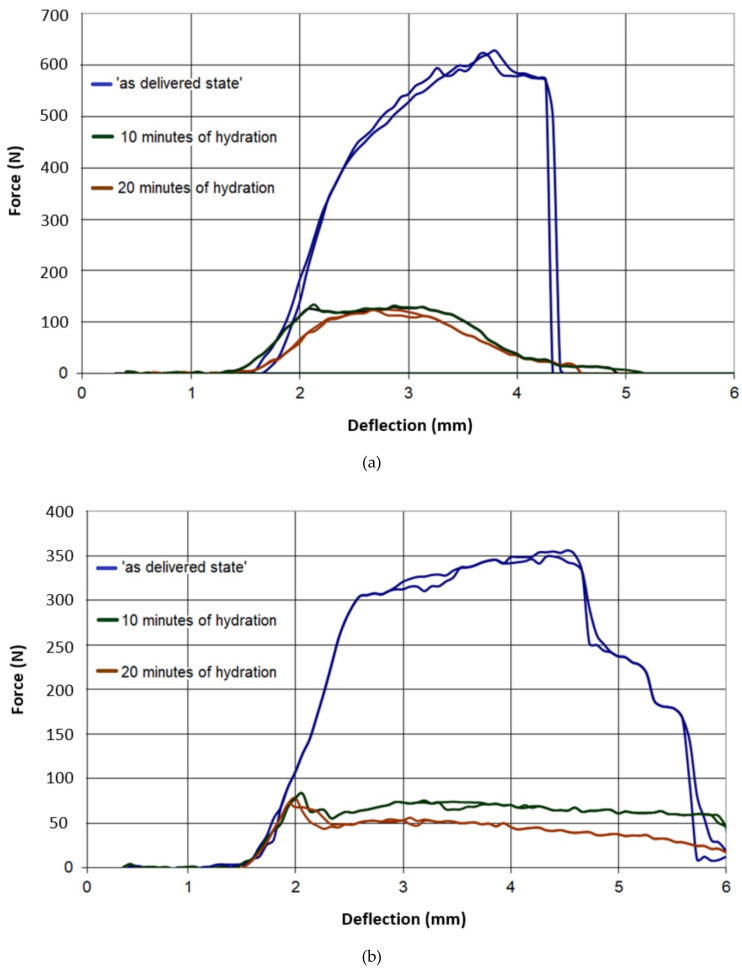
Force–deflection curves of the tested specimens of type B, (**a**) parallel to the sheet production direction, (**b**) perpendicular to sheet production direction.

**Figure 9:**
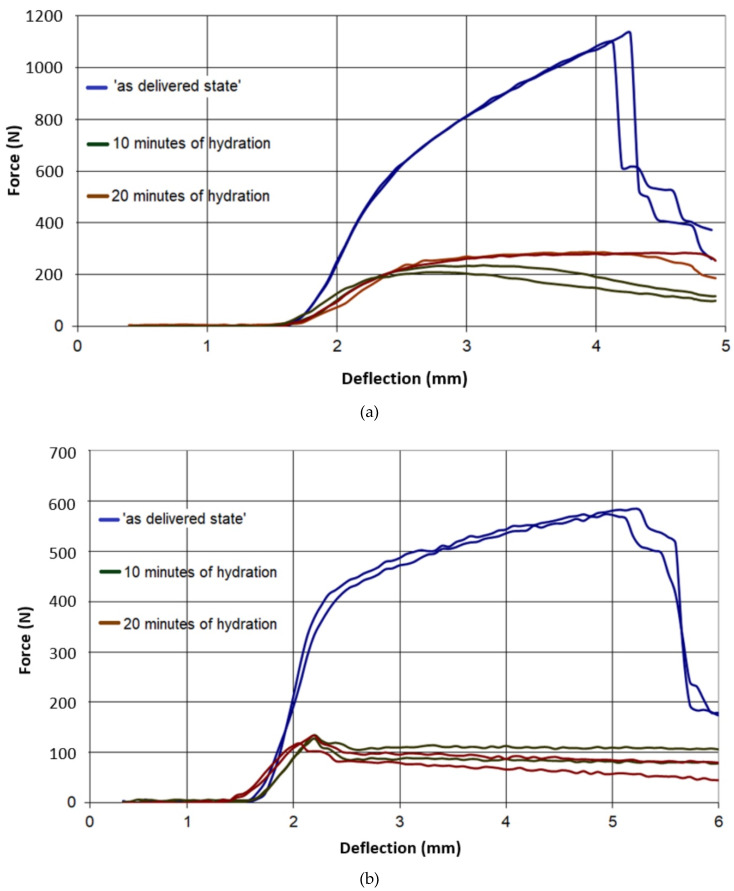
Force–deflection curves of the tested specimens of type G, (**a**) parallel to the sheet production direction, (**b**) perpendicular to sheet production direction.

**Figure 10:**
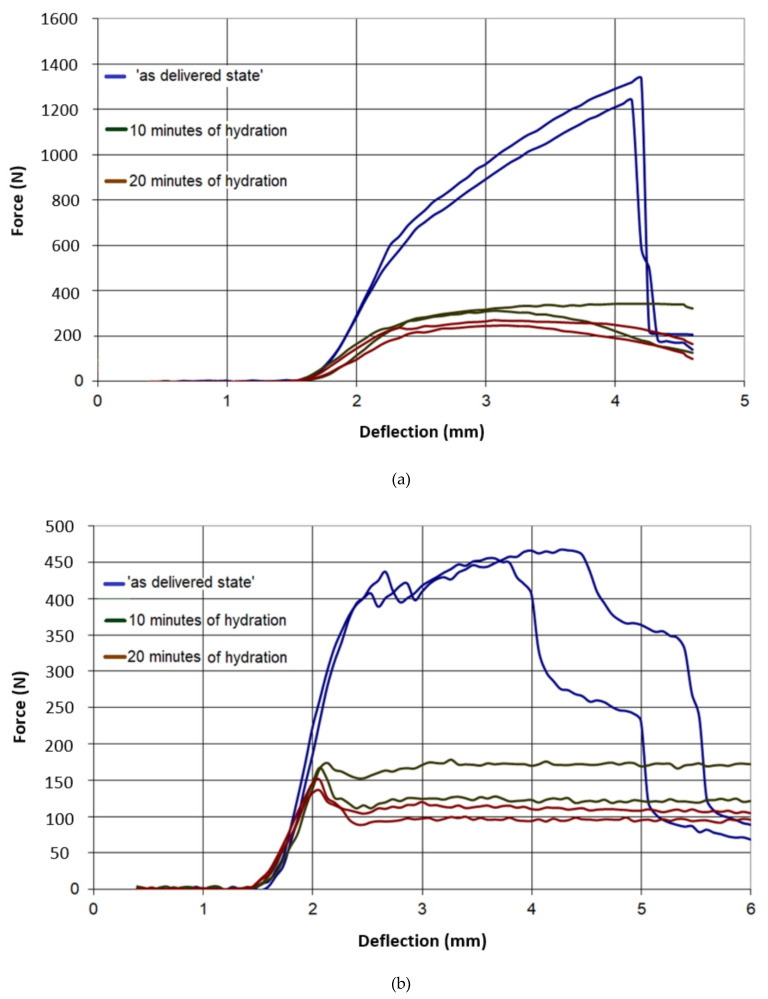
Force–deflection curves of the tested specimens of type I, (**a**) parallel to the sheet production direction, (**b**) perpendicular to sheet production direction.

**Figure 11:**
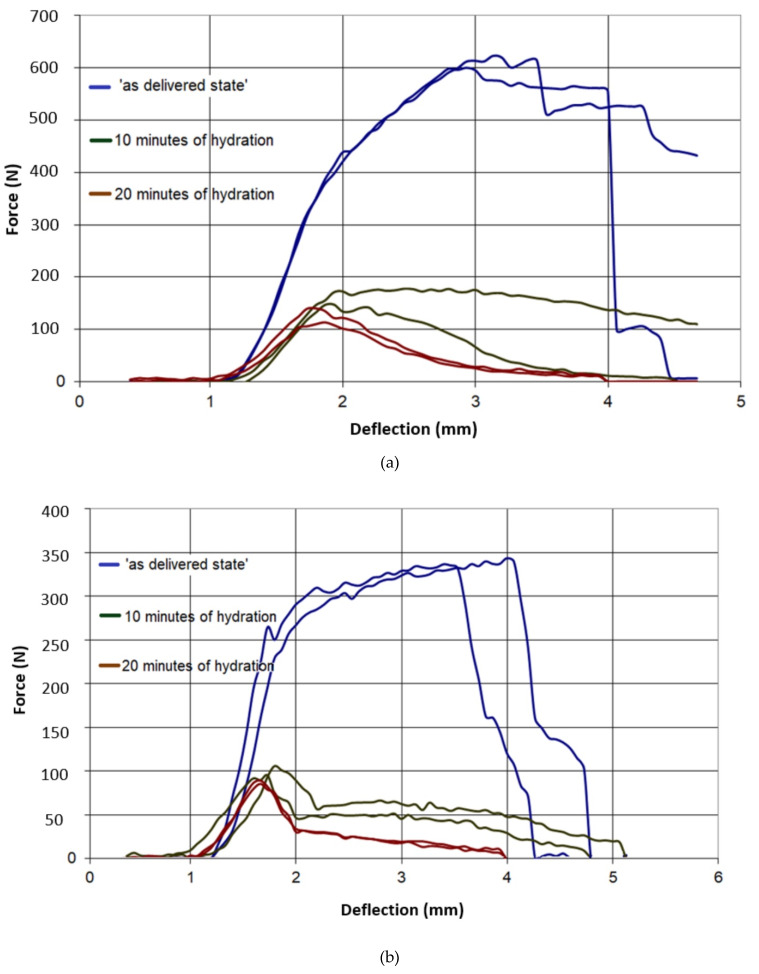
Force–deflection curves of the tested specimens of type K, (**a**) parallel to the sheet production direction, (**b**) perpendicular to sheet production direction.

The results of all three-point bending tests are presented in Figure 7, Figure 8, Figure 9, Figure 10 and Figure 11. In these figures, the blue curves represent the behavior of the specimens in the as-delivered state, the green ones—after first hydration cycle—and the brown curves—after two hydration cycles. The results were recalculated to determine the max. stress *S* [MPa] occurring in the central specimens’ cross sections, using the standard formula:

S = (3Fl)/(2bh^2^) (4)

*F* stands for the loading force, *l*—for the length of the support span, *b*—specimen width, *h*—specimen thickness there. The averaged results of all mechanical tests were listed in Table 4. 

The difference between the results of the mechanical tests obtained after the first and second hydration cycles was relatively small. However, the loss of mechanical strength after the first hydration was significant. Such loss may indicate that, after two hydration cycles, nearly all possibilities to introduce the moisture to the boards pore system were exhausted.

It was observed in Figure 7 and Figure 8 that both type A and B in the as-delivered condition have similar mechanical strengths. However, the B-type boards, characterized by higher open porosity system, were capable to absorb more moisture. Such absorption caused a higher loss of mechanical strength in both of the loading directions. The glass-fiber-reinforced boards of G and I type had a higher initial strength, as compared to different types of boards. However, the hydration process led to the deterioration of bond connection between the fibers and matrix and the subsequent loss of specimens’ strength (Figure 9 and Figure 10). The decrease in the mechanical properties for about 70%–80% was observed, regardless of the level of hydration. It was found that I and K types absorbed ca. 5% water by wt. and others (A,B) absorbed more than 30%.

### 3.3. Assessment of Ultrasonic Wave Velocity c_L_ Loss after Hydration Tests—Discussion

**Figure 12:**
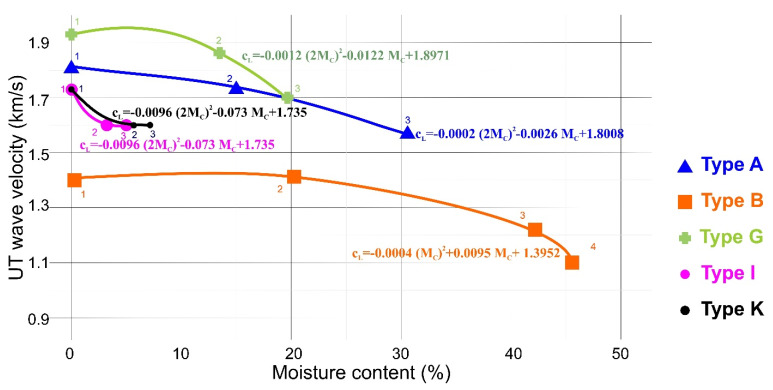
Results of the ultrasound testing (UT) tests. The UT wave velocity was measured in all specimens types before and after the hydration tests.

The results of UT tests are presented in Figure 12. For each plasterboard type, eight measurements at different board locations of accidental choice were performed and then the average wave velocity was determined. The standard deviation of a single series of measurements was included in the range of 2% ÷ 3% from the average value of the readings. The procedure was performed on the boards (1) in the as-delivered condition, (2) after immersing in water at room temperature for 10 min and 60 min of drying, and (3) after twice immersing for 10 min and 60 min of drying. These procedures were performed in order to obtain two states of the hydration, i.e., ‘the moderate hydration’ and ‘the strong hydtation’ in a sufficiently repetitive manner. Any intermediate state was not possible to achieve. It could be observed from the Figure 12, different values of c_L_ for different board types when the board humidity was zero. The results obtained during tests (2) and (3) take into account the loss of longitudinal wave velocity UT c_L_ in all types of analysis. The amount of this loss depended on the plate’s microstructure and varied from 6% to 35%. The accuracy of UT measurements was estimated as +/− 2% and could be further improved by performing more repetitions for each measurement. It is worth mentioning that the time of one series of UT measurements was estimated to 5 min, which makes a good recommendation for that method for the in-situ application.

Based on the results of the UT measurements, it was possible to propose four approximate formulas (Formulas (5)–(8)) to describe the UT wave velocity (km/s) dependence on the moisture content (%). The correlation coefficient calculated for these approximations was greater than 0.9. The testing of standard board (specimens of type A) showed that hydration procedures increase the material moisture content to 31%. Within that range of $M_{c}\;$, the following formula is valid:

(5) $$c_{L}=–\;0.0002\;{\left(2M_{c}\right)}^{2}–0.0026\;M_{c}+1.8008$$

The fire-resistant board exhibited a lower water permeability during the tests and thus UT wave velocity measurements were higher in this material. Therefore, within the range of $M_{c}\;$up to 19%, the following formula is valid for the specimens of type G:

(6) $$c_{L}=–\;0.0012\;{\left(2M_{c}\right)}^{2}–0.0122\;M_{c}+1.8971$$

Boards with a compact matrix of K and I types were least permeable for the humidity and they finally achieved about 5% of the moisture content after the tests. In this case, the following formula with increased linear term is proposed:

(7) $$c_{L}=\;0.0096\;{\left(2M_{c}\right)}^{2}–0.073\;M_{c}+1.735$$

The low-quality board (B) characterized by a greater contribution of open porosity exhibited the lowest value of UT wave velocity. The specific type of microstructure caused the highest increase in the material moisture content up to 47%. The theoretical model of influence of open and closed porosity on $c_{L}$ is more widely discussed in [25]. In that last case, quadratic polynomial function was unsuccessful due to the necessary function increase for small moisture content, which was not observed. Therefore, it is proposed to neglect the square term of the equation and apply the following formula:

(8) $$c_{L}=\;–0.0093\;M_{c}+1.4092$$

## 4. Conclusions

The effect of the hydration processes on the mechanical strength of five types of the porous gypsum plasterboards was investigated throughout this research. Three-point bending tests have revealed the strength loss of 70%–80% for all tested specimens due to hydration. Subsequent Ultrasonic (UT) measurements revealed that longitudinal wave velocity *c*_L_ decreased after hydration. Based on the plasterboard porosity volume [27], four types of approximated formulae were proposed in which the UT wave velocity (km/s) dependence on the moisture content (%) was described.

The dedicated UT transducers with low acoustic impedance and a solid-state polymer jelly interface were capable of achieving the required propagation parameters of UT waves and further determined their velocity in investigated plasterboard. The registered changes of c_L_ values were remarkably greater than the standard deviation of single series of measurements, included in the range of 2%÷3% from the average value of the readings. It was found that the ultrasonic method could be successfully used for plasterboards hydration processes monitoring. 

In authors’ opinions, more research work is required to determine the relationship between ultrasonic wave velocity and mechanical properties. That relationship strongly depends on the plasterboard type. For the standard indoor installation board type, it can be concluded that approx. a 70% loss of mechanical strength denotes a 10% loss of UT wave velocity. For I and K types, an 80% loss of mechanical strength denotes an 8% loss of UT wave velocity.

## Figures and Tables

**Table 1:** Mechanical properties of tested plasterboard compositions.

Board Symbol	Remarks	Board Thickness [mm]
A	standard board for indoor installations	12.5
B	low quality board of standard type	12.5
G	fire resistant type	12.5
I	acoustic insulating type	12.5
K	humidity resistant type	12.5

**Table 2:** Mechanical properties of tested plasterboard compositions (cont.).

Board Symbol	Apparent Density [kg/m^3^]	Flexural Strength F_max_ [N], Declared by the Manufacturer— Parallel and Perpendicular to the Sheet Length
A	648	>550/>210
B	648	**–**
G	880	>550/>210
I	1024	>550/>210
K	672	>550/>210

**Table 3:** Chemical composition of the plasterboards made of materials A and G.

wt. (%)	O	Si	S	Ca
A material	55.32	0.74	19.15	23.49
G material	56.54	1.20	19.52	22.74

**Table 4:** Results of the mechanical tests performed on investigated plasterboard types.

Board Type	Max. Stress in the Initial State, || to Sheet Production Direction [MPa]	Max. stress in the Initial State, |_ to Sheet Production Direction [MPa]	Max. stress after First Hydration, || to Sheet Production Direction [MPa]	Max. Stress after First hydration, |_ to Sheet Production Direction [MPa]	Max. Stress after Second Hydration || to Sheet Production Direction [MPa]	Max. Stress after Second Hydration |_ to Sheet Production Direction [MPa]
A	4.74	2.62	1.24	1.29	1.15	0.84
B	4.69	2.64	1.01	0.62	0.95	0.58
G	8.34	4.34	2.14	0.98	1.68	0.94
I	9.65	3.46	2.48	1.27	1.93	1.08
K	4.59	2.50	1.22	0.75	0.95	0.65

* The standard deviation of all obtained mechanical test results did not exceeded 5%.
